# Forsythoside A Inhibits BVDV Replication via TRAF2-Dependent CD28–4-1BB Signaling in Bovine PBMCs

**DOI:** 10.1371/journal.pone.0162791

**Published:** 2016-09-12

**Authors:** Quan-Jiang Song, Xiao-Gang Weng, Dong-Jie Cai, Wang Zhang, Jiu-Feng Wang

**Affiliations:** 1 Department of Veterinary Clinical Sciences, College of Veterinary Medicine, China Agricultural University, Beijing, China; 2 College of Life Science, Northeast Agricultural University, Harbin, Heilongjiang, China; Monash University, AUSTRALIA

## Abstract

Bovine viral diarrhea virus (BVDV), the causative agent of bovine viral diarrhea/mucosal disease (BVD/MD), is an important pathogen of cattle and other wild animals throughout the world. BVDV infection typically leads to an impaired immune response in cattle. In the present study, we investigated the effect of Forsythoside A (FTA) on BVDV infection of bovine peripheral blood mononuclear cells (PBMCs). We found that Forsythoside A could not only promote proliferation of PBMCs and T cells activation but also inhibit the replication of BVDV as well as apoptosis induced by BVDV. FTA treatment could counteract the BVDV-induced overproduction of IFN-γ to maintain the immune homeostasis in bovine PBMCs. At same time, FTA can enhance the secretion of IL-2. What’s more, BVDV promotes the expression of CD28, 4-1BB and TRAF-2, which can be modulated by FTA. Our data suggest that FTA protects PBMCs from BVDV infection possibly via TRAF2-dependent CD28–4-1BB signaling, which may activate PBMCs in response to BVDV infection. Therefore, this aids in the development of an effective adjuvant for vaccines against BVDV and other specific FTA-based therapies for preventing BVDV infection.

## Introduction

Bovine viral diarrhea virus (BVDV) is a member of the *Pestivirus* genus of the *Flaviviridae* family, with a worldwide distribution, and the cause of severe economic loss. BVDV infection can cause several cattle diseases, in the form of both transient or persistent infections, thought to be the reservior of viral transimission [1,2]. BVDV is a single-stranded positive-sense RNA virus, 12.3–12.5 kb in length, and contains one large open reading frame (ORF) flanked by 5’ and 3’ untranslated regions (UTRs). The large ORF encodes structural proteins and nonstructural proteins [3]. BVDV is divided into two distinct species: BVDV-1 and BVDV-2. Both species are categorized as cytopathogenic (cp) and noncytopathogenic (ncp) based on their effect in cultured cells [1,4]. The Oregon C24V strain of BVDV is utilized in this study and causes fever, leukopenia, virema, a decreased number of lymphocytes in calves, and cytopathic effects [5,6]

Since BVDV remains a serious concern, a significant amount of research has been performed with respect to vaccine development. The goal of vaccination is to activate both B and T cell-mediated immune responses against BVDV to lower the incidence of acute and persistent infections in a herd. As a result of the heterogeneity among BVDV strains, the infectivity of the fetus, and persistent infections, it is very difficult to achieve adaptive immune protection against BVDV infection. Both the modified live and killed vaccines may be efficacious under controlled conditions. However, they are not 100% effective in every individual. Additionally, several safety concerns have also been associated with the current vaccines [7]. One method of overcoming this issue is through the use of adjuvants. An adjuvant can have multiple beneficial effects, enhancing both the magnitude and quality of immune responses specific to the coadministered antigen [8]. Thus, an effective adjuvant for the BVDV vaccine is imperative to enhance the cellular immune responses for cattle to prevent BVDV infections. In addition, another option for controlling BVDV infection is the use of antivirals.

Forsythoside A (FTA) is a polyphenolic constituent of the Fructus forsythiae (Lian Qiao) that has anti-inflammatory, anti-oxidative, and antiviral functionality [9–11]. Moreover, FTA treatment in chicken cells prior to viral infection inhibited the replication of avian infectious bronchitis virus [12]. Therefore, we hypothesized that FTA may possess similar antiviral functionality against BVDV, which should be tested.

Peripheral blood mononuclear cells (PBMCs) from healthy adult cattle primarily consist of approximately 25–35% CD4^+^ T cells, 10–22% CD8^+^ T cells, and 28–35% B cells [13]. It has been shown that B cells, T cells and monocytes in bovine PBMCs as well as macrophages all can be infected by BVDV strains [14–16].

Costimulatory signaling molecules play a vital role in regulating the differentiation of various T cell subsets, as well as T cell activation, effector function, and survival. CD28 and cytotoxic T-lymphocyte antigen 4 (CTLA-4) are prototypal costimulatory molecules on cell surface. In addition, they interact with same ligands (i.e., CD80/86) on the surface of professional antigen-presenting cells (APCs), known to be critical for T cell activation and immune regulation [17].

Tumor necrosis factor receptors (TNFRs), including 4-1BB and OX40, synergize with T cell receptor (TCR)-CD3 signaling to promote cell cycle progression, the production of cytokines, and T cell survival [18]. Moreover, they can form a trimeric ligand to promote the recruitment of TNF receptor-associated factor (TRAF) adaptor protein. The association of TNFRs with different TRAF family members can modulate both pro-survival and pro-apoptotic signals [19]. Furthermore, through costimulation with 4-1BB, OX40 promoted T cell survival through upregulating anti-apoptotic factors, which includes Bcl-2 and Bcl-xL. Bcl-xL was a repressor of apoptosis which discriminated between pro-apoptotic Ca^2+^ signals and pro-survival Ca^2+^ signals, inhibiting the former but enhancing the latter [20]. In contrast, Bim is able to bind and modulate the anti-apoptotic Bcl-2 proteins to promote apoptosis [21].

In addition, 4-1BB and OX40 are both able to promote T cell survival by increasing the level of Bcl-xL via NF-κB signaling and decreasing the level of Bim via extracellular signal-regulated kinase (ERK) expression; however, OX40-deficient T cells failed to maintain Bcl-xL levels [22,23]. IL-2 is an important cytokine that induces the proliferation of responsive T-cells and binds to interleukin 2 receptor (IL-2R) which could be upregulated in response to cp BVDV strains [24].

It was also reported that TNFRs promoted the production of IFN-γ [18]. Moreover, IgG antibodies triggered antibody-dependent cellular cytotoxicity directed against malignant or virally infected cells to protect the host [25].

In the present study, we hypothesized that FTA affects the replication of BVDV, and is a viable candidate for use as an adjuvant in BVDV vaccines or other therapeutics. We therefore investigate the influence of FTA on the replication of BVDV and its effects on the apoptosis, proliferation and activation of PBMCs. In addition, the expression levels of costimulatory signals (CD28/CTLA-4), members of the TNFR family (4-1BB/4-1BBL, OX40) and related factors, including TRAFs and apoptosis-related factors in PBMCs infected with BVDV were evaluated to reveal possible mechanisms.

## Materials and Methods

### Ethics statement

All animal were treated in strict accordance with the *Guidelines for Laboratory Animal Use and Care from the Chinese Center* for Disease Control and Prevention, and the *Rules for Medical Laboratory Animals* from the Chinese Ministry of Health, under protocol CAU-AEC-2013-073 approved by the Animal Ethics Committee of the China Agricultural University.

### Virus and FTA

The C24V strain of BVDV was purchased from the China Veterinary Culture Collection Center (Beijing, China). Freeze-dried FTA was purchased from Dalian Biomedical Center (Dalian, China).

### Animals and blood sampling

Two-year-old Holstein cows (n = 6), free of BVDV-specific antibodies and antigens, were obtained from a commercial cattle farm. A 20 ml volume of peripheral blood was collected from the jugular vein and anti-coagulated with 2 ml of 2 × acid—citrate—dextrose solution (Sigma-Aldrich, St. Louis, MO).

### Isolation of bovine peripheral blood mononuclear cells

Peripheral blood mononuclear cells were isolated from the whole blood of the cows using the procedure described previously by Zhang [26]. Briefly, 10 ml of Ficoll solution (Sigma-Aldrich, St. Louis, MO) was stratified under 20 ml of peripheral blood and centrifuged at 400 × g for 20 min at room temperature. Then recovered PBMCs were washed three times with 5 volumes of Dulbecco’s PBS (Hyclone, Logan, Utah). The pellet was washed three times with RPMI 1640 medium (Gibco, Grand Island, NY) and centrifuged at 200 × g for 10 min at 4°C. Trypan blue (Sigma-Aldrich) staining was used to count the number of living cells at a concentration of 0.4 mg/ml under a light microscope (Olympus, Tokyo, Japan).

### Cell cultures and Trypan blue staining

Bovine PBMCs were cultured in RPMI–1640 culture medium (Gibco, Grand Island, NY) containing 2 mM L-glutamine enriched with 100 U/ml penicillin/streptomycin (Hyclone, Rockford, IL) and 10% heat-inactivated fetal bovine serum (Gibco). The cell concentration was adjusted to 2 × 10^6^ cells/ml in culture medium treated with BVDV (MOI 0.1) or FTA (100 μg/ml). Cells in the viral groups were divided into either non-FTA or FTA (100 μg/ml) treatment groups. Cells and supernatants were collected at 0, 12, 24, 48, 72, and 96 h after stimulation and stored at −80°C until further analysis. Trypan blue (Sigma-Aldrich) staining at a concentration of 0.4 mg/ml was used to count the number of living cells under a light microscope (Olympus, Tokyo, Japan) before collecting samples.

### Quantification of viral load in bovine PBMCs

Total RNA was extracted from cells bovine PBMCs using TRIzol reagent (Invitrogen). cDNA was synthesized using SuperScript III reverse transcriptase (Invitrogen) in a 20 μl reaction mixture. The 5'-UTR fragment of C24V was amplified and cloned into a simple pMD19-T vector (TaKaRa, Shiga, Japan). Inserts were sequenced to confirm the presence of the target fragment and 5-fold serial dilutions of plasmids encompassing the target fragments were used to establish the standard curves. The viral copies per cell were calculated based on the standard curve and living cell number. The following primer set was used: 5'-TAGTCGTCAGTGGTTCACGCC-3' and 5'-CCTCTGCAGCACCCTATCAG-3'.

### Extracellular infectivity titration

The infectivity titer was determined on MDBK cells by end-point dilution and immunofluorescence. Typically, 25 μl of supernatant was serially diluted tenfold in DMEM–10% FCS, and 100 μl was used to inoculate MDBK cells. Infection was examined at 72 h postinoculation by immunofluorescence, using BVDV 1&2 anti-E2 antibody (VMRD, Pullman, Washington) with the appropriate secondary tetramethyl rhodamine isothio-cyanate (TRICT)-conjugated antibody (Proteintech, Chicago, IL).

### CFSE assay

Bovine PBMCs (5 × 10^6^ cells/ml) were stained with 5(6)-Carboxyfluorescein diacetate N-succinimidylester (CFSE) (Invitrogen, Carlsbad, CA) at 0.5 μM in PBS/0.1% bovine serum albumin (BSA) for 10 min at 37°C in the dark. Afterwards, the reaction was quenched by the addition of ice-cold media with 10% fetal bovine serum to the cells. Stained cells were pelleted by centrifugation and washed three times by resuspending the pellet in fresh media. Bovine PBMCs were treated under one of five conditions, as follows: (i) medium; (ii) BVDV infection alone; (iii) FTA incubation alone; (iv) simultaneous incubation with BVDV plus FTA; and (v) incubation with phytohaemagglutinin (PHA) only at 10 μg/ml as positive control. Then the cells were cultured in round bottom 96-well plates. Finally, proliferation at 72 and 96 h after stimulation was measured by FACScalibur flow cytometer (Biosciences, Franklin Lakes, NJ)

### Quantitative real-time PCR

The total RNA was extracted from bovine PBMCs using TRIzol reagent (Invitrogen). The sequences of the primers used are listed in Table 1. To quantify relative mRNA expression, the cycle threshold (C_T_) values of the target genes were normalized to the C_T_ values of the reference β-actin gene, and the results were presented as fold change using the 2^−ΔΔCt^ method. The relative expression of target gene mRNA in each group was calculated using the following equations:ΔCT = C_T target gene_ − C_T β-actin gene,_ and ΔΔCT = ΔC_T treated group_ − ΔC_T control group_.

**Table 1:** Primers used for real-time PCR, length of the respective PCR product and gene accession number.

Gene product	Primer		Product length (bp)	GenBank number
	Direction^a^	Sequence (5’-3’)		
CD28	F	GGAGGTCTGTGCTGTGAATGG	70	NM_181004.1
	R	GGTGCAGTTGAATTCCTTATTT		
CTLA-4	F	TGCCCGGATTCTGATTTTCTCCTC	146	NM_174297.1
	R	GGCATTTTCACATAGACCCCTG		
4-1BB	F	AATGATCAGGAACATGGCATC	284	NM_001035336.2
	R	AGCTTCTTTCTGCCCTGCTT		
4-1BBL	F	TGCCCTTGAGATAGTTCACC	156	XM_005198935.3
	R	TGCTGCTTCCCCTAGTGT		
OX40	F	AGCCGCTGTAACCACAACCA	222	NM_001099043.2
	R	GCAGTCAACTCCACGCTT		
TRAF-1	F	TGTTCGTCAAGTGCGTCGTG	297	NM_001192801.1
	R	CAGTTCTGCGTGGTGAGTGC		
TRAF-2	F	AAGAAGATCGCCCGTGAGAAG	87	XM_869007.6
	R	GCAGCCAACAGCGTGGAAT		
Bcl-xL	F	CGACGGGCATTCAGCGACCT	122	XM_015474116.1
	R	GCCACAATGCGACCCCAGTTCACC		
Bim	F	AGCCCGGCACCCATGAGTTGT	215	XM_010809718.2
	R	GCCTGGTGACGCACGAAGACCCT		
β-actin	F	CAAGGAGAAGCTCTGCTACG	232	NM_173979.3
	R	GATGTCGACGTCACACTTCA		

^a^ F = forward; R = reverse.

### Concentrations of IFN-γ, IL-2 and IgG2a

Concentrations of IFN-γ, IgG2a and IL-2 in the cell culture supernatants were determined using commercially available ELISA kits specific for bovine IFN-γ (Bethyl, Montgomery, TX), bovine IL-2 (Hiton, Tianjin, China) and mouse IgG2a (Abcam, Cambridge, UK). The ELISAs were performed according to the manufacturer’s instructions.

### Western blotting

PBMCs were pelleted and lysed in Radio-Immunoprecipitation Assay (RIPA; 50 mM Tris-HCl, pH 8.0, 150 mM sodium chloride, 1.0% Nonidet P-40, 0.5% sodium deoxycholate, 0.1% sodium dodecyl sulfate) buffer (Sigma-Aldrich) supplemented with 1 mM phenylmethanesulfonyl fluoride (Amresco, Solon, OH). After vortexing briefly, the lysates were incubated on ice for 10 min and centrifuged to remove the insoluble pellet. The protein concentration in the supernatant was determined using a BCA protein assay kit (CoWin Biotech, Beijing, China). The primary antibodies used were goat anti-human 4-1BB affinity purified monoclonal antibody (AF838, R&D, Minneapolis, MN), rabbit anti-CD28 polyclonal antibody (Bioss, Shanghai, China), goat anti-TRAF2 polyclonal antibody (Abcam, Cambridge, UK), mouse anti-BVDV E2 monoclonal antibody (our lab prepared) and mouse anti-glyceraldehyde-3-phosphate dehydrogenase ([GAPDH], 60004-1-Ig) (Proteintech, Chicago, IL). The peroxidase-conjugated affinipure donkey anti-goat IgG(H+L) (10285-1-AP, Proteintech, Chicago, IL), Horseradish peroxidase (HRP)-conjugated affinipure goat anti-rabbit IgG(H+L) (SA00001-2, Proteintech, Chicago, IL) and HRP-conjugated affinipure goat anti-mouse IgG(H+L) (SA00001-1, Proteintech, Chicago, IL) were used as the secondary antibodies. GAPDH served as an internal control and exhibited stable expression regardless of treatment. Results are presented as the ratio of target protein band intensity to the GAPDH band intensity.

### Apoptosis assay

Apoptosis of PBMCs was assessed using an apoptosis kit with fluorescein isothiocyanate (FITC)-conjugated annexin V and propidium iodide (PI) for flow cytometry (Vazyme, Nanjing, China). At 24 h after different treatment, PBMCs were harvested, washed in pre-chilled PBS, and stained with FITC-conjugated annexin V (5 μl) and PI (5 μl) in succession for 10 min at room temperature. Appropriate single-labeled and unlabeled controls were included. After filtering through a 70-μm nylon cell strainer, cells were assessed for fluorescence using a FAC Scalibur flow cytometer (BD Biosciences) equipped with FlowJo software. The percentages of early apoptotic (annexin-FITC-positive/PI-negative) and late apoptotic (annexin-FITC/PI-double positive) cells were analyzed.

### Flow cytometry

To assess T cell activation, the percentage of CD3^+^CD25^+^ was analyzed by flow cytometry at 12 h after different treatments. The PBMCs treated with 2 μg concanavalin A (ConA) were treated as positive control. The following antibodies were used: Alexa Fluor 647-conjugated rat anti-human CD3 (Bio-Rad, Hercules, CA) and isotype control Alexa Fluor 647 conjugated-rat IgG1 (BD Biosciences) FITC-conjugated mouse anti bovine CD25 (GeneTex, San-Antonio, TX) and isotype control mouse FITC-conjugated mouse IgG1 (BD Biosciences)

For each reaction, 2 × 10^6^ cells were suspended in 50 μl of 0.1% bovine serum albumin (BSA)-PBS (wash buffer) and stained and cells were selected by gating based on size and granularity to exclude other cells and cellular debris. At least 1 × 10^5^ gated events per condition were acquired.

### Statistical analysis

Data were analyzed using the MIXED procedure of SAS version 9.2 (SAS Institute Inc., Cary, NC). Differences between the lowest square means were compared using a Student’s t-test and Tukey’s test. A *P*-value of < 0.05 was considered statistically significant.

## Results

### Effect of FTA on the replication of BVDV in bovine PBMCs

**Fig 1:**
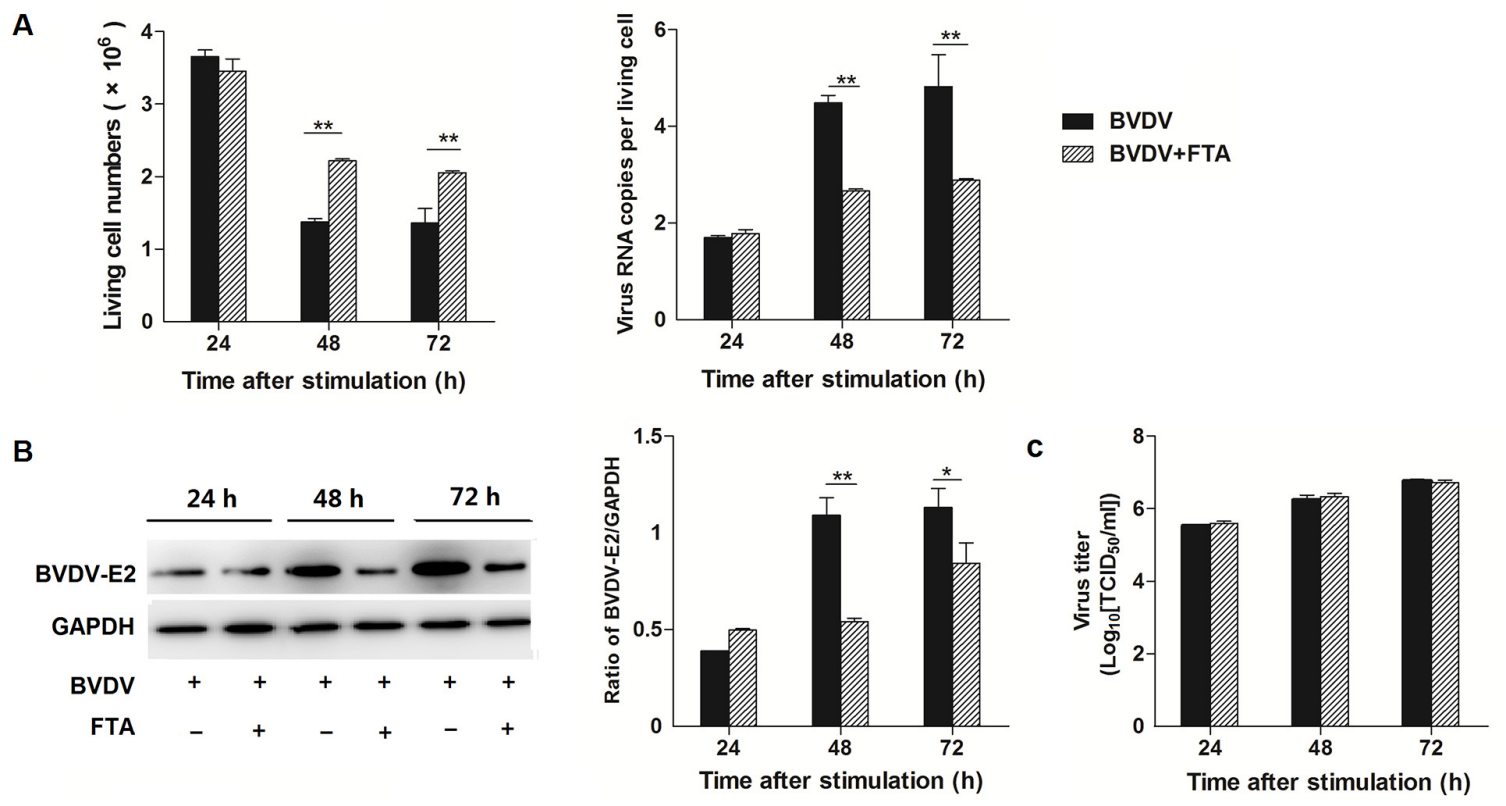
Effect of FTA on BVDV replication and virion infectivity. (A) The number of living cell (left panels) and virus copies per cell (right panels) at 24, 48, and 72 h after simulation. The number were counted with Trypan blue stain and the virus copies per living cells were measured by absolute quantitative real-time PCR for amplifying the 5’ UTR. (B) The BVDV-E2 protein express by western blot (left panels) and ratio of E2 band intensity to that of GAPDH (right panels). BVDV E2 protein in PBMCs was collected from the indicated PBMCs cultures at 24, 48, and 72 h after stimulation. Expression of GAPDH was measured as an internal control. (C) BVDV titers in supernatants of PBMCs treated with BVDV or BVDV and FTA at 24, 48, and 72h. Data are presented as the means ± SEM of three independent experiments. *P < 0.05, **P < 0.01, ***P < 0.001.

To investigate the effect of FTA on the replication of BVDV, the numbers of living cells was counted with Trypan blue staining and copies of BVDV RNA were quantified using absolute quantitative real-time PCR (Fig 1A). There were more living cells in PBMCs treated with BVDV plus FTA than in cells only infected with BVDV at 48 and 72 h after stimulation (*P* = 0.001 and *P* = 0.006, respectively). At 24 h post incubation with BVDV, there was no difference observed in the number of BVDV RNA copies between PBMCs stimulated with FTA plus BVDV and BVDV alone. However at 48 and 72 h post incubation with BVDV, the copies of BVDV RNA per cell treated with BVDV plus FTA decreased compared with the cell only infected with BVDV (*P* = 0.003 and *P* = 0.001, respectively; Fig 1A).

At 48 and 72 h after BVDV infection, BVDV E2 protein expression was lower in PBMCs treated with BVDV plus FTA than in cells only infected with BVDV (*P* = 0.001 and *P* = 0.045, respectively; Fig 1B).

### BVDV titers in supernatants of PBMCs

To investigate the virion infectivity, the virus titers were measured in supernatants of PBMCs treated with BVDV or BVDV with FTA after 24, 48 and 72 h infection. Our results showed that FTA had no effect on BVDV titers in the supernatants (Fig 1C).

### Effect of FTA on the proliferation of PBMCs

**Fig 2:**
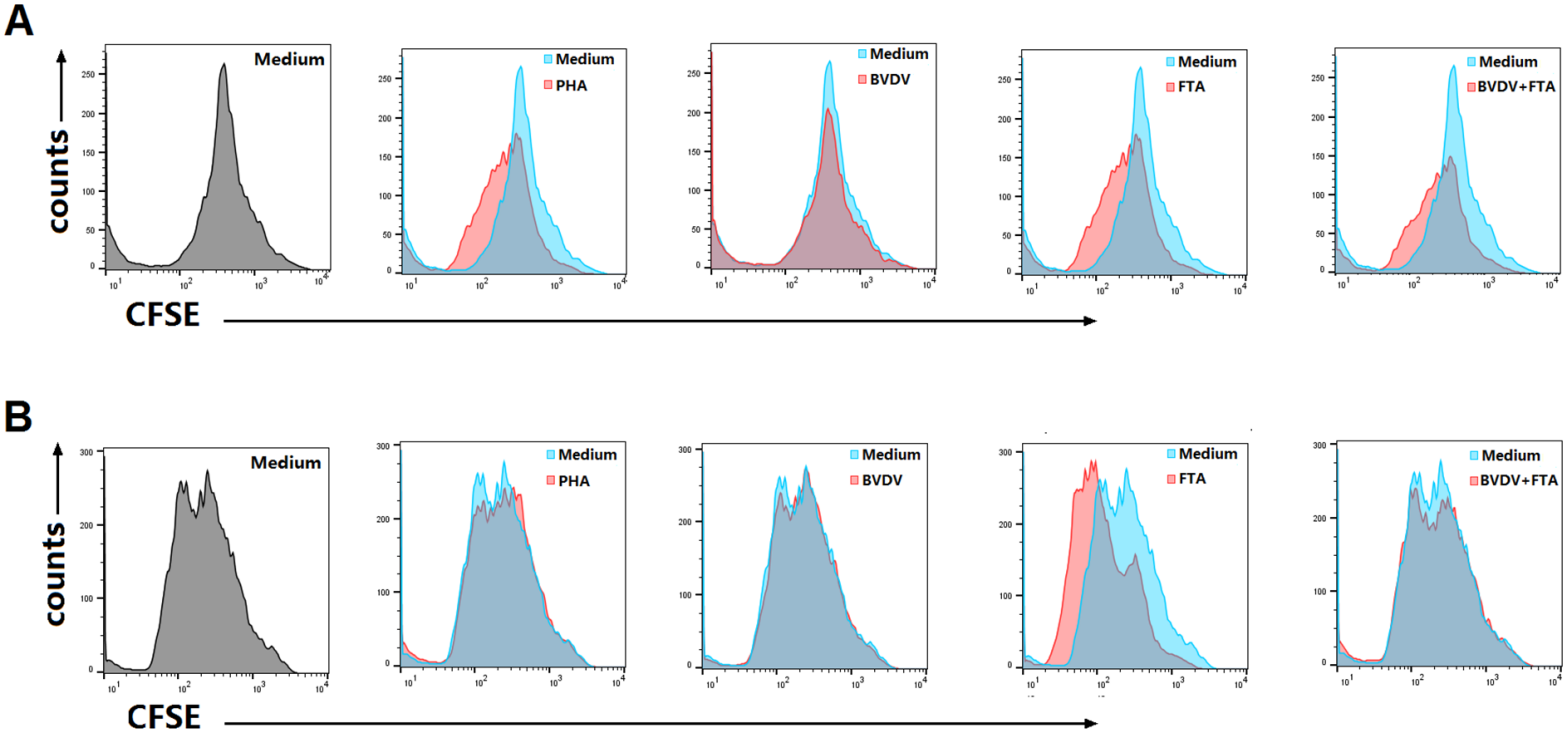
Proliferation of PBMCs after stimulation. Bovine PBMCs were labeled with CFSE before stimulation with FTA alone, BVDV alone or BVDV plus FTA, and were assessed for their ability to proliferate at 72 (A)and 96 h (B) later by flow cytometry.

To investigate the effect of FTA on the proliferation of PBMCs, a CFSE assay was performed. CFSE-stained bovine PBMCs were cultivated in the presence of FTA, BVDV and PHA or medium alone for three days before being analyzed by flow cytometry. Compared with the medium, the bovine PBMCs treated with FTA alone or BVDV plus FTA proliferated apparently at 72 h (Fig 2A) and 96 h (Fig 2B) after stimulation.

### Effect of FTA on costimulatory molecules and members of the TNFR family in bovine PBMCs

To understand the effect of FTA on costimulatory molecules in bovine PBMCs following BVDV challenge, the relative mRNA expression for genes of selected costimulatory molecules and members of the TNFR family (CD28, CTLA-4, 4-1BB, 4-1BBL, and OX40) was quantified.

**Fig 3:**
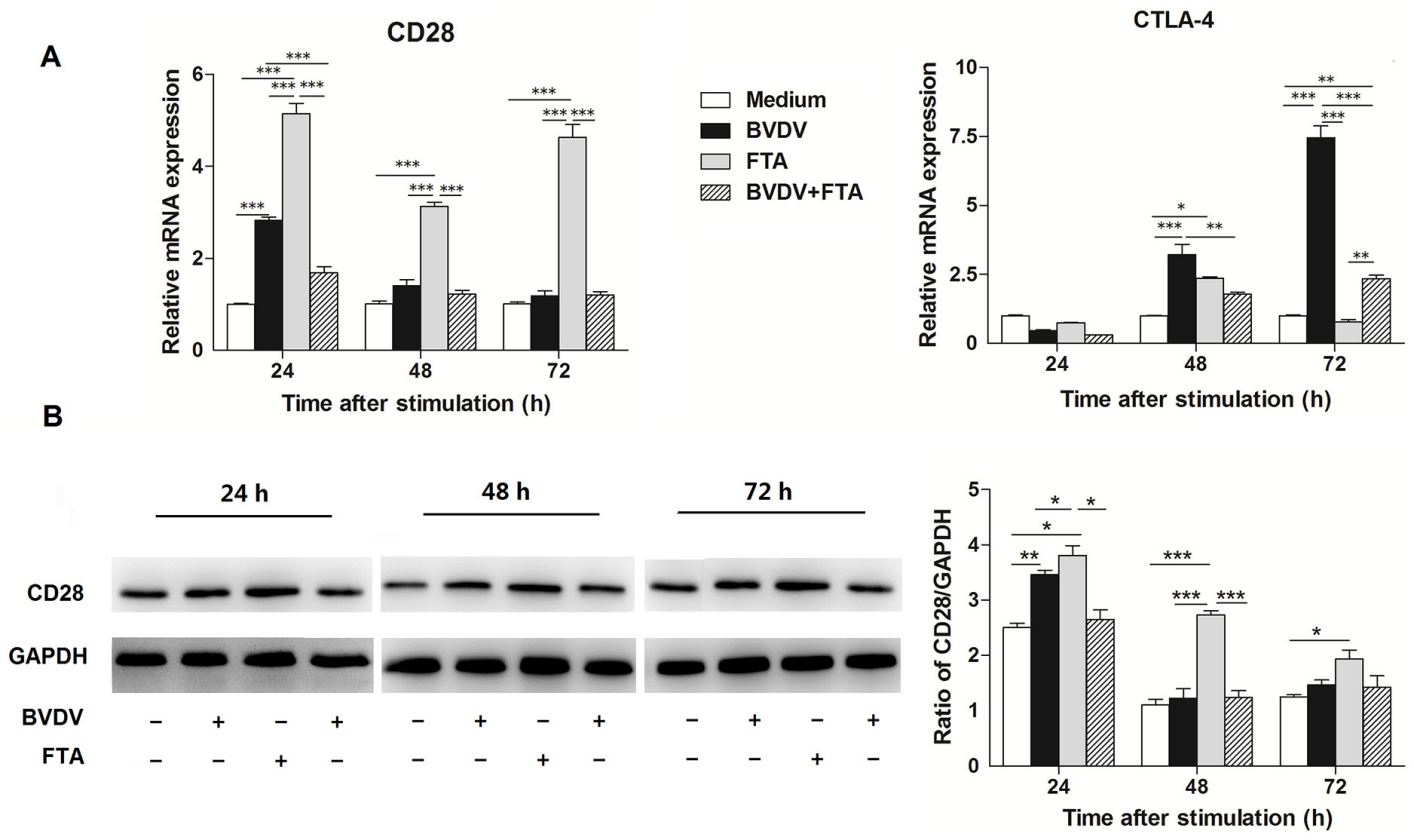
Effect of FTA on the expression of CD28 and CTLA-4 in bovine PBMCs infected with BVDV. (A) Relative mRNA expression of CD28/CTLA-4 cultured with medium alone, FTA, BVDV, and BVDV plus FTA at 24, 48, 72 h in bovine PBMCs. (B) The CD28 protein express by western blot (left panels) and ratio of CD28 band intensity to that of GAPDH (right panels). CD28 protein in PBMCs was collected from the indicated PBMCs cultures at 24, 48, and 72 h after stimulation. Expression of GAPDH was measured as an internal control. Data are presented as the means ± SEM of three independent experiments. *P < 0.05, **P < 0.01, ***P < 0.001.

At 24, 48, and 72 h after stimulation, there was an increase in CD28 mRNA expression in the PBMCs stimulated with FTA alone (*P* < 0.0001), but not in those treated with BVDV plus FTA compared to the untreated controls. However, an increase in CD28 mRNA expression was observed in the PBMCs stimulated with BVDV alone compared with controls 24 h after stimulation (*P* = 0.0003; Fig 3A).

An increase in CTLA-4 mRNA expression was observed in PBMCs infected with BVDV alone, but not the cells treated with FTA alone or BVDV plus FTA compared to the controls at 48 and 72 h after infection (*P* = 0.0002 and *P* < 0.0001, respectively; Fig 3A).

Similar to the mRNA expression, western blot analysis revealed that there was also an increase in CD28 protein expression at 24 h after BVDV challenge in bovine PBMCs (*P* = 0.014; Fig 3B). Consistently, CD28 protein expression was higher at 24, 48, and 72 h in PBMCs treated with FTA alone than in the control cells (*P* = 0.009, *P* < 0.0001, and *P* = 0.035, respectively).

**Fig 4:**
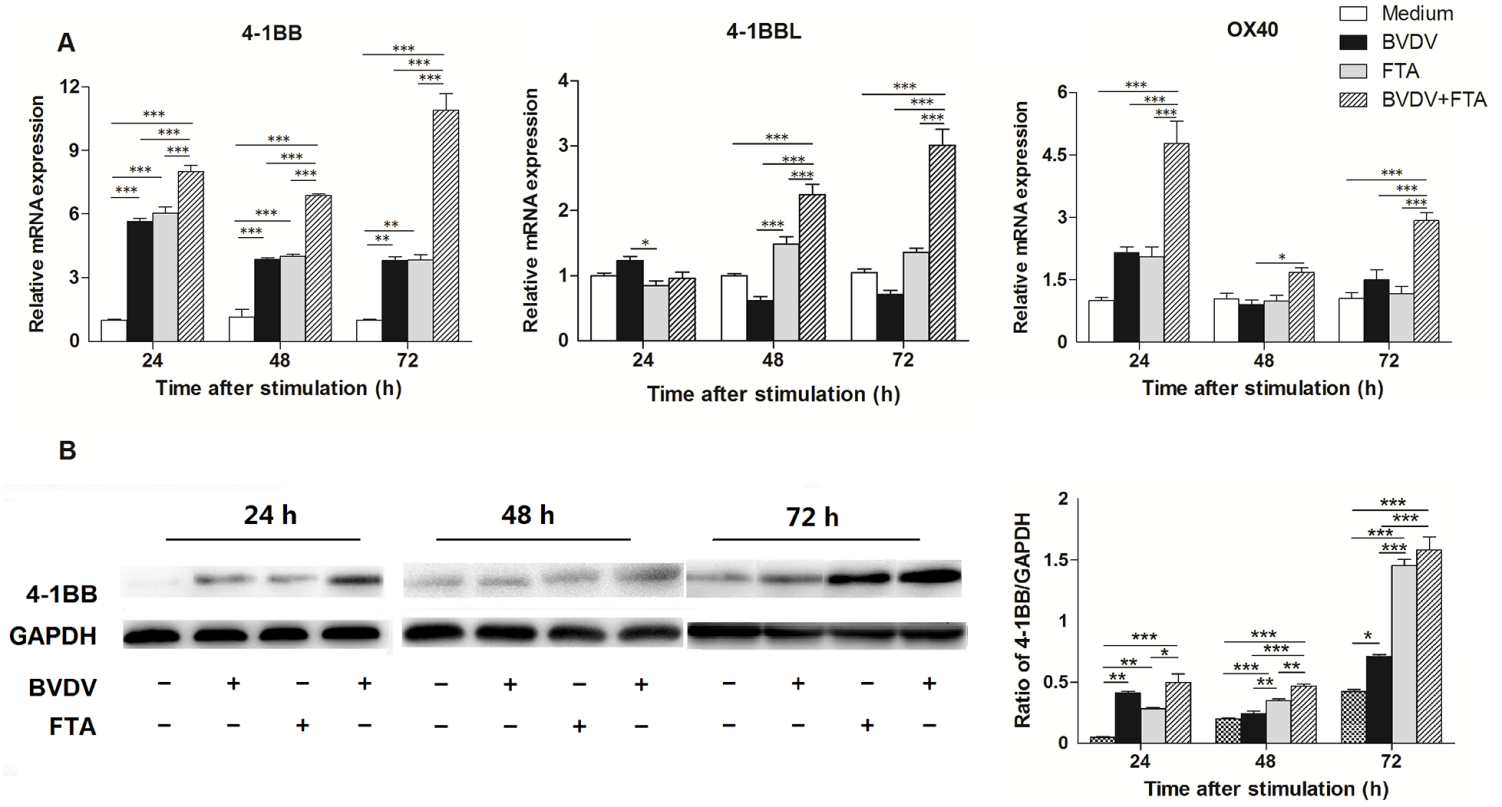
Effect of FTA on the expression of OX40, 4-1BB, and 4-1BBL in bovine PBMCs infected with BVDV. (A) Relative mRNA expression of OX40, 4-1BB, and 4-1BBL cultured with medium alone, FTA, BVDV, and BVDV plus FTA at 24, 48, 72 h in bovine PBMCs. (B) The 4-1BB protein express by western blot (left panels) and ratio of 4-1BB band intensity to that of GAPDH (right panels). 4-1BB protein in PBMCs was collected from the indicated PBMCs cultures at 24, 48, and 72 h after stimulation. Expression of GAPDH was measured as an internal control. Data are presented as the means ± SEM of three independent experiments. *P < 0.05, **P < 0.01, ***P < 0.001.

Compared with the untreated cells, there was an increase in 4-1BB mRNA expression in the cells only infected with BVDV and treated with FTA alone, as well as BVDV plus FTA at 24, 48, and 72 h after stimulation (Fig 4A). At 48 and 72 h after stimulation, there was an increase in 4-1BBL mRNA expression in the PBMCs stimulated with BVDV plus FTA, but not the cells only infected with BVDV or those treated with FTA alone compared with untreated controls (*P* = 0.0002 and *P* < 0.0001, respectively). Compared with the untreated control cells, in the presence of BVDV plus FTA, the expression of OX40 mRNA in PBMCs was increased at 24 and 72 h following stimulation (*P* < 0.0002 and *P* = 0.0006, respectively).

Moreover, the western blot analysis also revealed that 4-1BB protein expression was increased in the cells only infected with BVDV 24 and 72 h (*P* = 0.0001, *P* = 0.038, respectively; Fig 4B). There was an increase in 4-1BB protein expression in PBMCs treated with BVDV plus FTA in comparison with the control cells at 48 and 72 h (*P* < 0.0001). It was higher in cells treated with BVDV plus FTA than in cells only infected with BVDV at 24, 48 and 72 h (P = 0.008, *P* < 0.0001 and *P* < 0.0001, respectively).

### The effect of FTA on adaptor molecules

**Fig 5:**
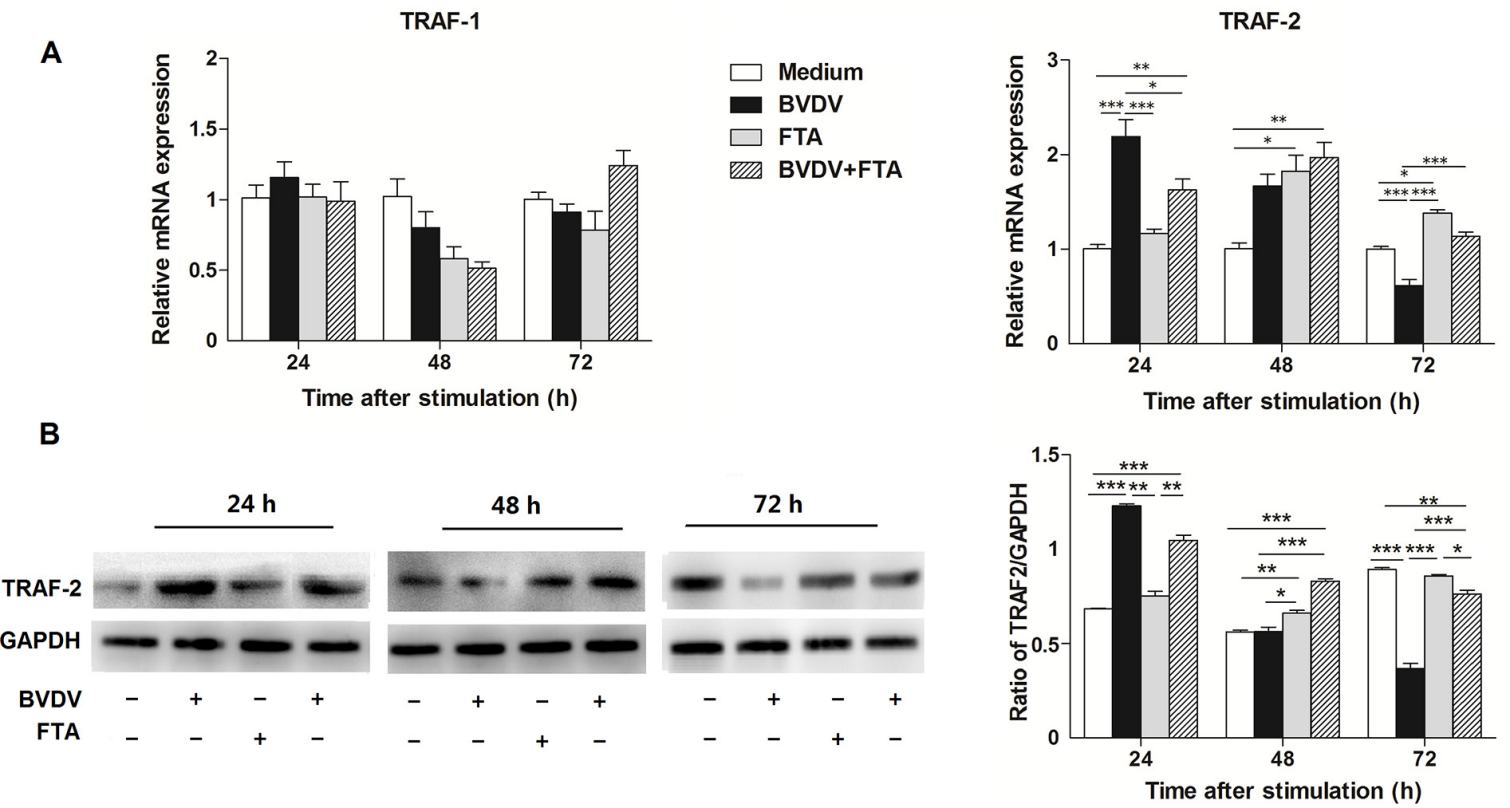
Effect of FTA on the expression of TRAF1 and TRAF2 in bovine PBMCs infected with BVDV. (A) Relative mRNA expression of TRAF-1 and TRAF-2 cultured with medium alone, FTA, BVDV, and BVDV plus FTA at 24, 48, 72 h in bovine PBMCs. (B) The TRAF-2 protein express by western blot (left panels) and ratio of TRAF-2 band intensity to that of GAPDH (right panels). TRAF-2 protein in PBMCs was collected from the indicated PBMCs cultures at 24, 48, and 72 h after stimulation. Expression of GAPDH was measured as an internal control. Data are presented as the means ± SEM of three independent experiments. *P < 0.05, **P < 0.01, ***P < 0.001.

To investigate the effect of FTA on the adaptor molecules in PBMCs after BVDV challenge, the expression of mRNAs of selected adaptor molecules (i.e., TRAF1, TRAF2) were quantified. There were no differences in TRAF1 mRNA expression among the different groups at 24, 48, or 72 h (Fig 5A). Compared with the untreated cells, TRAF-2 mRNA expression increased (*P* < 0.0001) at 24 h but decreased (*P* = 0.0008) at 72 h after infection with BVDV. By comparison, the TRAF-2 mRNA expression increased at 48 h (*P* = 0.0264) and 72 h (*P* < 0.0001) after stimulation with FTA. The expression of TRAF-2 mRNA in PBMCs stimulated by BVDV plus FTA was higher than the untreated cells at 24 and 48 h (*P* = 0.0079 and *P* = 0.0058, respectively).

Similar to the mRNA expression results, western blot analysis revealed that TRAF-2 protein increased (*P* < 0.0001) at 24 h but decreased (*P* < 0.0001) at 72 h after BVDV challenge (Fig 5B). The TRAF-2 protein in PBMCs treated with BVDV plus FTA was higher than the untreated cells at 24 and 48 h (*P* <0.0001), but lower at 72 h (*P* = 0.003)

### Concentrations of IFN-γ, IL-2 and IgG2a in bovine PBMCs

**Fig 6:**
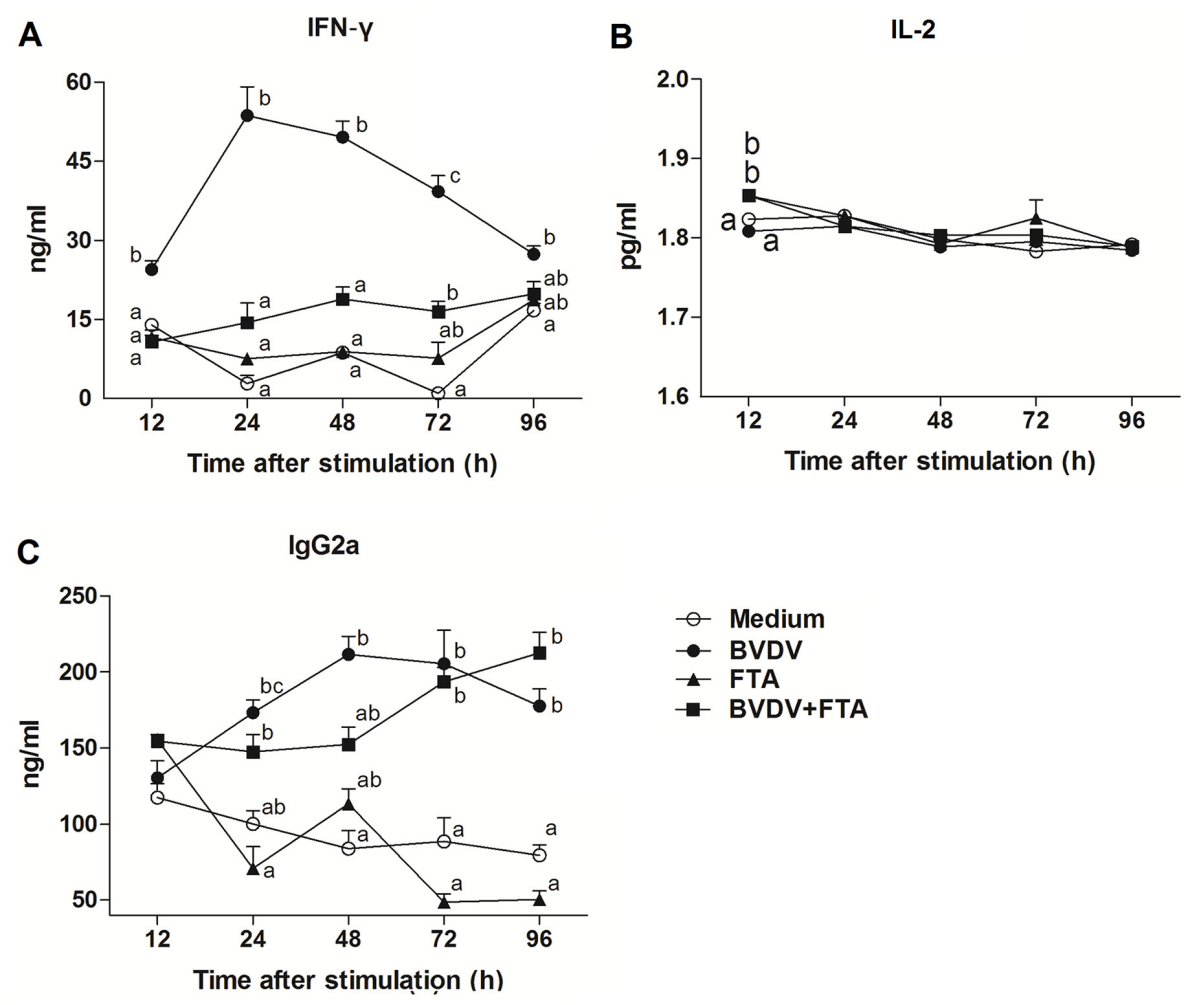
Concentrations of IFN-γ, IL-2, and IgG2a in cell culture supernatants. Bovine PBMCs cultured with medium alone, FTA, BVDV, and BVDV plus FTA. Concentrations of IFN-γ (A), IL-2 (B) and IgG2a (C) were detected by ELISA. Data are presented as means ± SEM of three independent experiments. Mean values at the same time point without a common superscript (^a, b, c^) differ significantly (P <0.05).

Compared with untreated control cells, the concentrations of IFN-γ in the cell culture supernatants were increased in the presence of BVDV alone from 24 h after infection but not in the presence FTA alone. The concentrations of IFN-γ in the cell culture supernatants were increased in the presence of the concentrations of IFN-γ in the cell culture supernatants were higher in the presence of BVDV plus FTA alone at 72 h after infection compared with the untreated cells (*P* = 0.0115, Fig 6A).

Compared to the control, concentration of IL-2 was higher in supernatants of PBMCs treated with FTA alone and FTA plus BVDV (*P* = 0.006 and *P* = 0.006, respectively; Fig 6B) at 12 h after stimulation.

The concentrations of IgG2a were higher both in the presence of BVDV alone from 24 h (*P* = 0.0253, *P* = 0.0004, *P* = 0.0038, and *P* = 0.0003, respectively) and BVDV plus FTA from 72 h (*P* = 0.0117 and *P* < 0.0001) compared to the medium controls (Fig 6C).

### Effect of FTA on apoptosis of bovine PBMCs

**Fig 7:**
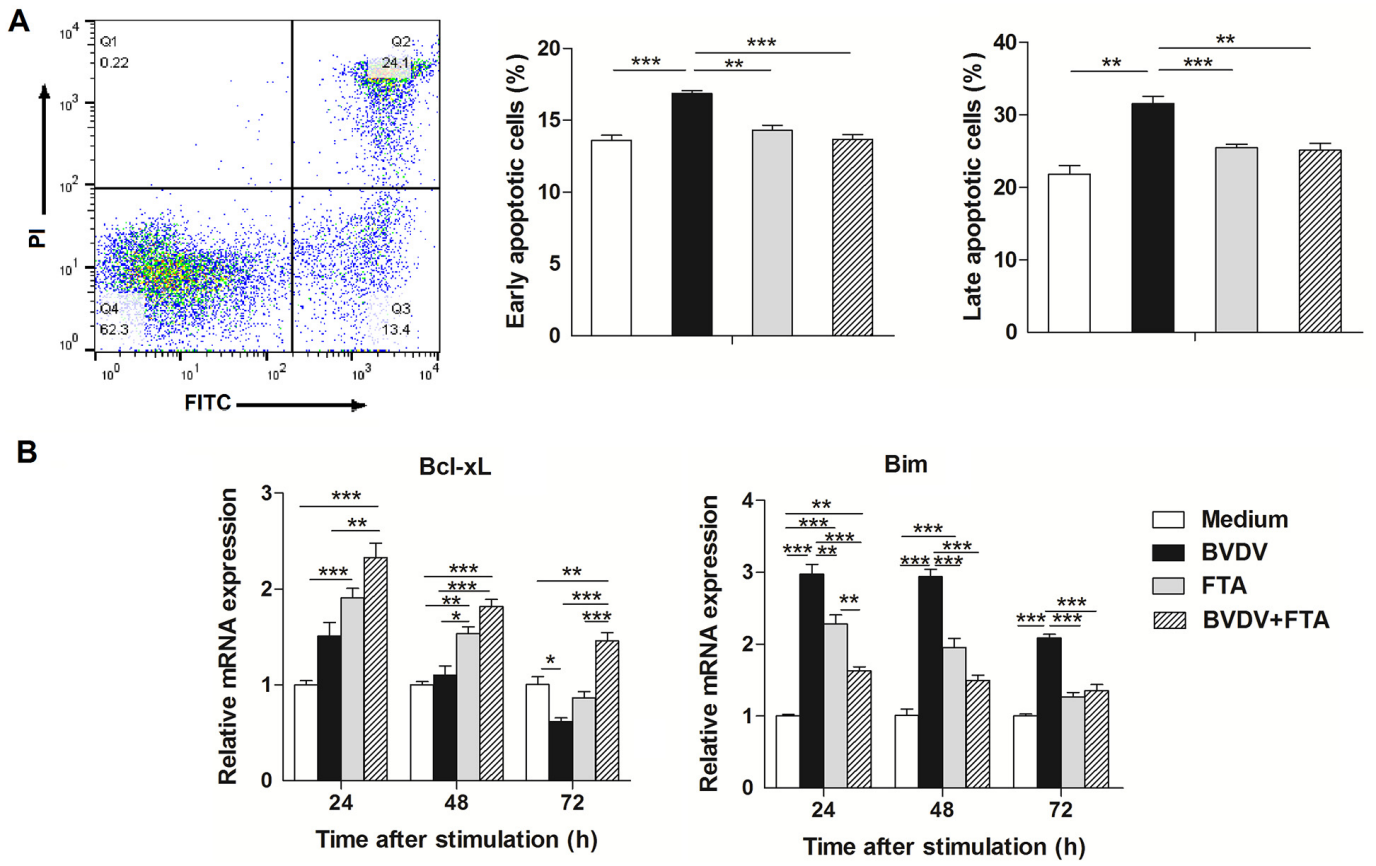
Effect of FTA on apoptosis of PBMCs infected with BVDV. PBMCs were collected from the indicated cultures at 24 h after BVDV challenge. (A) Representative two-dimensional scatter plots of annexin V versus propidium iodide, the percentage of early apoptotic cells and the percentage of late apoptotic cells. (B) Relative mRNA expression of Bcl-xL and Bim in bovine PBMCs after simulation. Data are presented as means ± SEM of three independent experiments. *P < 0.05, **P < 0.01, ***P < 0.001.

Bovine PBMCs exposed to BVDV alone had a higher percentage of early and late apoptosis compared with the untreated PBMCs (*P* < 0.001). FTA resulted in a decrease in the percentage of early and late apoptosis during BVDV infection (*P* < 0.0001 and *P* = 0.006, respectively; Fig 7A).

There was an increase in Bcl-xL mRNA expression in the presence of FTA only at 24 and 48 h (*P* = 0.0005 and *P* = 0.0011, respectively) but a decrease in cells only infected with BVDV at 72 h after infection (*P* = 0.0371; Fig 7B). Bim mRNA expression was increased in PBMCs only infected with BVDV (*P* < 0.0001, *P* < 0.0001 and *P* < 0.0001, respectively) compared to the controls. There was a decrease in the levels of Bim mRNA in cells treated with BVDV plus FTA at 24 h (*P* = 0.0037) compared to the controls (Fig 7B).

### Effect of FTA on T cell activation

**Fig 8:**
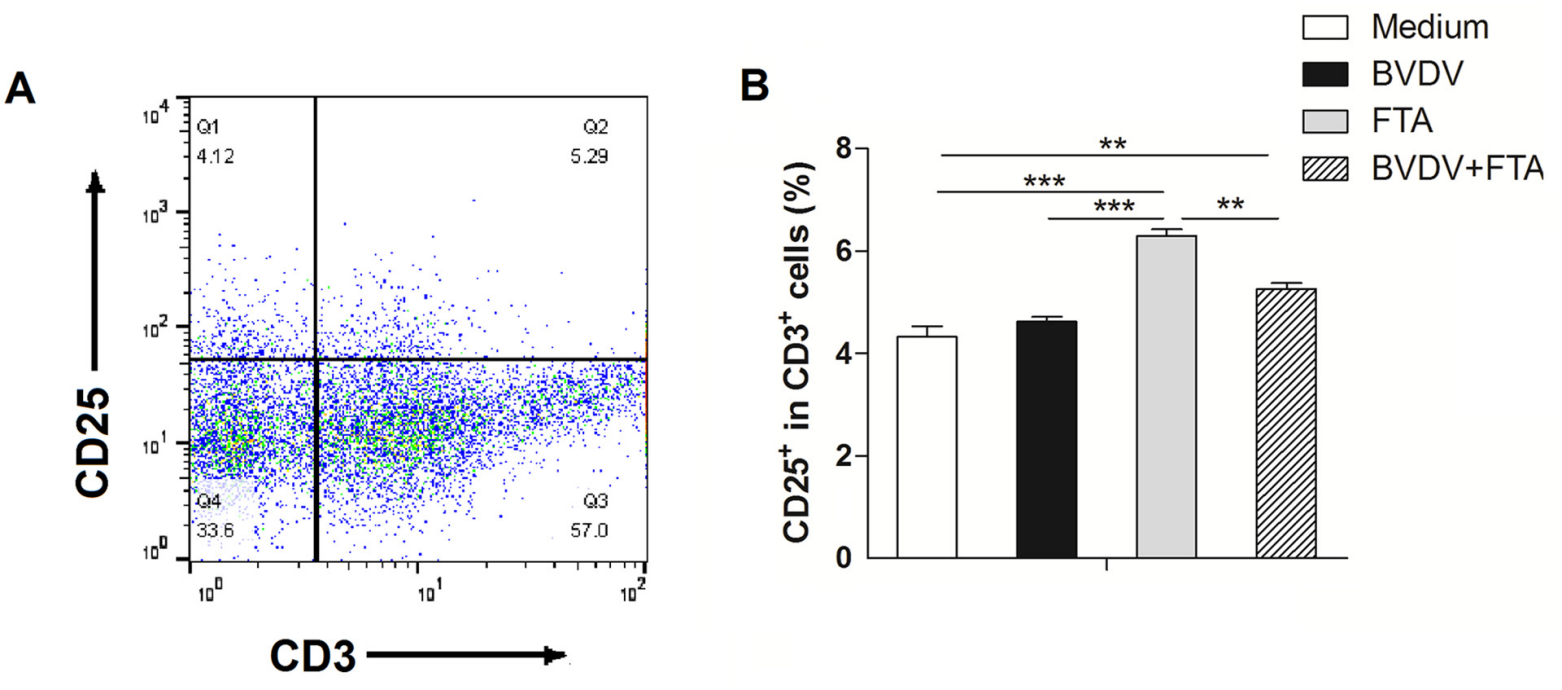
Effect of FTA on activation of T cells during BVDV infection. PBMCs were collected at 12 h after stimulation. (A) Representative flow cytometry dot plots, (B) Flow cytometry was used to determine the percentage of CD3^+^CD25^+^ population in the bovine PBMCs. Data are presented as means ± SEM of three independent experiments. *P < 0.05, **P < 0.01, ***P < 0.001.

The percentage of CD3^+^CD25^+^ cells was used to evaluate the condition of T cell activation. Compared with the medium controls, the percentage of CD3^+^CD25^+^ cells was increased both in PBMCs treated with FTA alone and in cells treated with BVDV plus FTA, but not in PBMCs only infected with BVDV (*P* < 0.0001 and *P* = 0.007, respectively; Fig 8).

## Discussion

A series of experiments were carried out to verify the immunomodulatory effects of FTA in PBMCs infected with BVDV. In this study, we demonstrate that FTA can inhibit the replication of BVDV and the apoptosis of PBMCs induced by BVDV. FTA also can promote the proliferation of PBMCs and activation of T cells. In addition, FTA could regulate the expression of the CD28 and 4-1BB, as well as downstream signaling events in PBMCs. Moreover, FTA can modulate the secretion of IFN-γ and IL-2 induced by BVDV.

In order for FTA to be used effectively, a number of unknown factors must be determined. First, the dose of FTA should be adjusted according to the respective virulence of each viral strain. In addition, the absorption rate of FTA should be increased. This can be achieved using chitosan derivatives within the intestinal mucosa to improve the absorption and bioavailability of FTA [27]. In the present study, FTA exhibited the potential capability of inhibiting the replication of BVDV.

Consistent with a previous study [28], the increase of virus titers in supernatants with infection time and the increased expression of BVDV-E2 indicated that BVDV could replicate in PBMCs. According to the number of living cells, FTA promotes PBMCs survival. The increase of living cell numbers may be the results of its effects of FTA on pro-proliferation and anti-apoptosis. Notably, FTA inhibits replication of BVDV in PBMCs. Consistent with our results, FTA displayed a direct virucidal effect in the study of infectious bronchitis virus *in vitro* [12]. FTA had a major impact only on decreasing intracellular BVDV RNA and protein levels but did not decrease virion infectivity, suggesting that replication of the BVDV strain used in this study is sensitive to FTA and FTA during BVDV infection did not result in a deleterious action. Activating the CD28/CD80 signal in hepatitis B virus transgenic mice was effective in promoting activated natural killer T (NKT) cell function to inhibit hepatitis B virus (HBV) replication by increasing the production of IFN-γ [29]. Moreover, 4-1BB stimulation had a striking effect on the induction of T cells to suppress the viral load [30]. Therefore, our findings indicated that FTA may inhibit the replication of BVDV through CD28—4-1BB signaling.

CD28 is a homodimeric stimulatory cell surface receptor of the Ig superfamily and its signaling is associated with antimicrobial immunity [31]. In naive T cells, signaling through TCR and CD28 induces proliferation, IL-2 production and protects from apoptosis [32]. Mice deficient in CD28 show an array of immune defects, including impaired T cell activation, a lack of T cell help for B cells, and poor memory T cell responses. All of these effects highlight the importance of CD28 costimulation in the generation of effective T cell responses and immune memory [33]. Engagement of CD28 leads to tyrosine phosphorylation of its cytoplasmic region and recruitment of cytoplasmic signaling proteins [34]. In this study, BVDV promoted the upregulation of CD28 mRNA and protein expression during the initial stage of infection. However, the expression of CD28 mRNA in the spleen and tracheobronchial lymph nodes (TBLN) did not change in the BVDV-inoculated groups by five days post infection *in vivo*, regardless of a low (strain SD-1) or high (strain 1373) virulence ncp BVDV [35]. Accordingly, the differential expression of CD28 might reflect differences in immune activation induced by these viral strains, which could be associated with differences in biotypes of BVDV.

Cytotoxic T lymphocyte antigen (CTLA)-4, a coinhibitory molecule, contributes to immunological tolerance and negative regulation of the immune response [36]. Similarly, our previous study demonstrated that classical swine fever virus also upregulated CTLA-4 expression in porcine PBMCs [37]. CTLA-4 revealed its viral-protective effect as inhibitory molecules that suppressed cytotoxic T-cells and thereby prevented the destruction of virus-infected hepatocytes in symptomatic acute hepatitis [38]. Furthermore, a blockade of one or more coinhibitory receptors (e.g., CTLA-4) can enhance virus-specific T cell function in hepatitis C virus infection [39]. In addition, the CD28/CTLA-4 was influenced by the expression of two shared ligands, CD80/CD86 [33]. Additionally, CTLA-4 prevented the induction of the integrin-mediated “stop signal” in T cells in contact with APCs. This “stop signal” contributed to the formation of a stable immunological synapse induced by T cell receptor ligation, and was likely amplified by CD28 co-ligation [40]. Moreover, the period in which CD28 is lower and less responsive to signaling is the same time during which CTLA-4 is upregulated in the present study. This indicates that the function of CTLA-4 may be active at a time when the CD28 function is impaired. Therefore, CD28/CTLA-4 is perhaps an integrated system to control costimulatory signals and their downstream effects during BVDV infection. FTA exhibited the ability to alleviate the suppression of cytotoxic T-cells induced by CTLA-4 in bovine PBMCs infected with BVDV. Further research is needed to determine how the interaction between BVDV, CD28/CTLA-4, and FTA regulates the balance between T cell activation and self-tolerance during BVDV infection.

Members of the TNFR family play key roles in regulating the magnitude, duration, and immune phenotype in response to viral infection [41]. OX40 is rapidly upregulated following T cell activation, most prominently on activated CD4^+^ T cells. Costimulation via OX40 exerts broad effects on the overall T cell immunity In physiological immune responses, OX40 delivers a potent costimulatory signal to activated CD4^+^ T cells, which supports their survival and proliferation. OX40 can bind with TRAF-2 activate both the canonical NF-κB1 pathway as well as the noncanonical NF-κB2 pathway [42]. The antigen load or extent of viral replication might affect the length of time that OX40 is on a specific T cell subset in response to viral infection [43]. Thus, the BVDV load may be not sufficient to activate OX40 in the present study.

4-1BB (CD137) is a member of the costimulatory TNFR family primarily expressed on activated T cells, and preferentially regulates the expansion and differentiation of CD8^+^ T cells. 4-1BB is primarily expressed on activated T cells and plays crucial roles in enhancing cytotoxicity of T cells, upregulating survival-related genes, and producing Th1 cytokines such as IL-2 and IFN-γ [44]. 4-1BB stimulation has also been shown to have a striking effect on the induction of T cells to suppress viral load [30]. It was reported that the effect of 4-1BB/4-1BBL on T cell numbers followed CD28 signaling [45]. A novel finding was that circulating CD4^+^CD28^null^ T cells from acute coronary syndrome patients expressed higher levels of 4-1BB costimulatory receptors compared to classical CD4^+^CD28^+^ T lymphocytes [46]. Similarly, the protein expression of 4-1BB was not entirely consistent with the expression of CD28 in BVDV infection in the present study. 4-1BB expression is induced by TCR ligation and the cross-linking of CD28 [18]. Thus, FTA might regulate 4-1BB expression in BVDV-infected bovine PBMCs through synergizing the signals of both the TCR and CD28.

The duration of 4-1BBL expression was linked to the persistence of viral antigen presentation. In a study of influenza, the duration of 4-1BB expression was sustained for longer periods when infected with severe strains of influenza virus than mild strains, indicating that 4-1BB/4-1BBL might be connected with the control of the viral load [23]. In the present study, the expression of 4-1BBL also varied with infection time, which may be related to the persistence of viral antigen presentation. Therefore, FTA has the potential ability to upregulate the expression of 4-1BBL during BVDV infection.

Due to the lack of intrinsic enzymatic activity, the TNFR family members rely on the recruitment of TRAFs to induce downstream signaling [47]. TRAF1 plays a pivotal role in adjusting T cell activation both by limiting the costimulation-independent NF-κB-inducing kinase (NIK) activation, and through facilitating the 4-1BB-induced classical NF-κB pathway of activation [48]. In our study, no alteration of TRAF1 expression following stimulation with BVDV or FTA was observed. TRAF1 might not activate downstream signaling pathways, due to the lack of an array of zinc finger domains and a RING finger domain, which is able to potentially function as an E3 ubiquitin ligase [47]. It has been reported that human immunodeficiency virus (HIV) was able to reduce the expression level of TRAF1 in HIV specific CD8^+^ T cells. Additionally, the loss of TRAF1 was correlated with an increased viral load [49]. TRAF2 was recruited by endocytosed 4-1BB—containing vesicles, and 4-1BB mediated NF-κB activation was inhibited by the enforced expression of a TRAF2 mutant lacking RING and zinc finger domains [50]. Thus, TRAF2 was potentially involved in BVDV-induced CD28–4-1BB signaling.

In the present study, FTA can suppressed early and late apoptosis induced by the C24V strain of BVDV. It was reported with the infection of 1373 strain of BVDV resulted in apoptosis of B cells in the lymphoid follicles in Peyer’s patches within 6 days following infection [16]. Our results revealed that the suppression may be related to upregulation of Bcl-xL and downregulation of Bim induced by FTA

4-1BB signaling increase T cell survival via the NF-κB-dependent upregulation of Bcl-xL and ERK-dependent downregulation of Bim [23]. OX40 promotes T cell survival by facilitating Bcl-xL expression and OX40-deficient T cells fail to maintain Bcl-xL levels [22]. Furthermore, TRAF2 has been shown to interact with caspase-8 downstream of Cullin3 by mediating RING-dependent, K48-linked polyubiquitination of the large catalytic domain of caspase-8 to inhibit apoptosis [51]. In addition, the alternation trend of TRAF2 mRNA was consistent with Bcl-xL during BVDV infection. Thus, the C24V strain of BVDV may induce the apoptosis of PBMCs. FTA may possess an apoptosis-inhibiting capacity by upregulating Bcl-xL expression and downregulating Bim expression. Thus FTA suppressed the BVDV-induced apoptosis of PBMCs through TRAF2 dependent CD28—4-1BB signaling.

IL-2 is an important cytokine that induces the proliferation of responsive T-cells [24]. Ncp BVDV did not boost the secretion of IL-2 in PBMCs of cattle [52,53]. However, cp BVDV strains increased IL-2R receptor expression [52], which was similar to our results. It was reported that CD28 costimulation of human and murine γδ cells induced secretion of IL-2 required for proliferation [54]. The ligand of CD28, CD80 also can cosimulate IL-2 production by T cells [34]. What’s more, 4-1BB triggering markedly increased IL-2Rα (CD25) and IL-2 expressions of CD8^+^ T cells. Thus, the increased production of IL-2 induced by FTA may be the result of CD28–4-1BB signaling. FTA may promote the bovine PBMCs proliferation via increasing secretion of IL-2 induced by CD28 and 4-1BB signaling [24,44].

Proliferating CD8^+^ lymphocytes produce IFN-γ indicating type 1 memory response in BVDV seropositive cattle [24]. IFN-γ promotes CD8^+^ T cell expansion and memory formation [55,56]. On the other hand, it also induces the contraction of the effector CD8^+^ T cell pool via promoting caspase-8-dependent apoptosis [57]. Thus, agonistic and antagonistic effects of IFN-γ must be balanced to generate functional CD8^+^ T cell responses during infection [58]. In the present study, the overproduction of IFN-γ caused by BVDV infection was confined by the addition of FTA, indicating that FTA regulates IFN-γ secretion and has an immunomodulatory effect on cellular immunity to maintain homeostasis. In addition, IFN-γ signal could promote increase expression of costimulatory molecules in APC which in turn facilitates initiation of the adaptive immune response against viral infection [59]. Similarly, an increase of IFN-γ level was observed in ncp BVDV-1 infected animals, suggesting that IFN-γ plays a key role in antiviral defense against ncp BVDV-1 infection, therefore prevents the development of pathological effects [24]. Moreover, IFN-γ can induce apoptosis eliminate virus infected cells [59]. Hence the apoptosis induce by BVDV may be partly due to increased IFN-γ.

Complete activation of T cells required both the engagement of the TCR with MHC/Ag and costimulation signals [60]. CD25 is one of late T cell activation makers [61]. CD28 interactions with the B7 family of costimulatory ligand are essential for initiating antigen-specific T cell responses, upregulating cytokine expression and promoting T cell expansion and differentiation [62]. CD25 is also the IL-2 receptor α subunit, which can be upregualted by the increased IL-2 [24]. In the present study, FTA can facilitate the activation of T cells, which may result from its regulation on CD28–4-1BB signaling and IL-2.

IgG2a can initiate antibody-dependent cellular cytotoxicity and kill influenza-infected cells [63]. However, for many autoimmune diseases, an intravenous infusion of high doses of pooled IgG may be an effective anti-inflammatory treatment [64]. In cattle, the ncp BVDV induced a CD4^+^ Th-2 response resulting in high levels of total BVDV-specific antibodies [53]. Therefore, FTA may exert its antiviral functions by facilitating the secretion of IgG2a.

In conclusion, our data indicate that FTA protects bovine PBMCs from BVDV infection, partly through inhibiting the replication of BVDV and the apoptosis of PBMCs as well as promoting the activation of T cells. The antiviral mechanism may be related to TRAF2-dependent CD28–4-1BB signaling, through which FTA attenuates the over secretion of IFN-γ caused by BVDV and boosts the secretion of IL-2 for PBMCs survival. Our findings suggest that FTA could be an effective adjuvant in vaccines against BVDV or other therapeutic strategies for preventing BVDV infection.
